# The Impact of Radiotherapy on the Primary Tumor in Patients with Metastatic High-Volume Castration-Sensitive Prostate Cancer: A Propensity Score Matching Analysis

**DOI:** 10.3390/cancers17020297

**Published:** 2025-01-17

**Authors:** Antoni Skripai, Philip Blumenfeld, Aaron Krakow, Robert Den, Aron Popovtzer, Marc Wygoda, Tal Falick Michaeli

**Affiliations:** 1 Department of Radiation Oncology, Sharett Institute of Oncology Hadassah Medical Center, Jerusalem 91120, Israel; antoni.skripai@mail.huji.ac.il (A.S.); aaron.krakow@mail.huji.ac.il (A.K.); robert.den@jefferson.edu (R.D.); mwygoda@hadassah.org.il (M.W.); 2 Department of Military Medicine and “Tzamert”, Faculty of Medicine, Hebrew University of Jerusalem, Jerusalem 91120, Israel; 3 Medical Corps, Israel Defense Forces, Ramat Gan 52625, Israel; 4 Department of Developmental Biology and Cancer Research, Institute for Medical Research Israel-Canada, Hebrew University Medical School, Jerusalem 91120, Israel

**Keywords:** metastatic prostate cancer, radiotherapy, external beam radiation therapy, propensity score matching, high metastatic burden

## Abstract

**Background:** Retrospective studies suggest that local radiotherapy on the prostate improves overall survival in the metastatic setting, but its benefit in patients with high-burden metastatic disease is still uncertain. **Methods:** We conducted a retrospective study of 100 high-metastatic-burden prostate cancer patients at Hadassah Ein Karem Medical Center from 2004 to 2021. Patients receiving local RT alongside standard treatment were compared to those receiving standard treatment alone. Prostate RT involved EBRT to the prostate/seminal vesicles with/without pelvic lymph nodes, delivering 54–60 Gy in up to 3 Gy fractions. The standard treatment used was ADT including or excluding chemotherapy. Subsequently, we performed 1:1 propensity score matching on 50 patients for further analysis. **Results:** The cohort had 100 patients split evenly between the RT and NRT groups. The RT group had higher Overall Survival than NRT (*p* < 0.046), remaining significant after Propensity Score Matching (PSM) (*p* < 0.034). Biochemical progression-free survival was also higher in RT vs. NRT (*p* < 0.033), maintaining significance after PSM (*p* < 0.042). RT patients reported longer symptom-free time vs. NRT (*p* < 0.017), though this difference did not persist post-PSM (*p* < 0.249). Post-PSM analysis showed higher loco-regional progression-free survival in the RT group vs. NRT (*p* < 0.049). **Conclusions:** Our study shows improved overall survival in the high-metastatic-burden group when adding RT to the standard treatment of choice, which persists after propensity score matching. The RT group in the entire cohort also demonstrated a reduction in progressing urinary symptoms and biochemical progression-free survival. These findings highlight radiotherapy’s potential as a viable treatment in these patients. However, prospective studies with comprehensive data collection are needed to fully address this topic.

## Simple Summary

Metastatic prostate cancer with high metastatic burden is challenging for treatment. Based on existing literature, it remains unclear whether external beam radiation therapy on the prostate can bring prognostic benefit in this scenario. In this retrospective cohort study using propensity score matching, we compared 50 patients who were treated with standard hormonal and chemotherapy treatments to 50 patients who, in addition to standard treatments, received external beam radiation therapy on the prostate. We found that patients who received radiotherapy had longer overall survival and longer biochemical progression-free survival. These results suggest that adding radiotherapy to the treatment plan may improve survival and other outcomes. Further research should be performed to investigate if this treatment approach is broadly applicable.

## 1. Introduction

Prostate cancer is the most common cancer as well as the third leading cause of death among men [1]. In 2024, an estimated 299 thousand new cases will have been diagnosed in the US [2]. Of these, 4% have de novo metastatic disease, and about one-third with locoregional disease will eventually develop metastasis [2]. The 5-year survival rate for metastatic prostate cancer in the US is approximately 32 percent [2], highlighting the need for improved therapies. In Israel, 3351 men were diagnosed with prostate cancer in 2021 and 525 men died from the disease, with a total mortality rate of 6% [3]. In a global comparison, Israel has a relatively high incidence rate of prostate cancer, with the mortality rate being relatively lower [3].

Surgery and radiation are the primary treatment of choice for localized prostate cancer, whereas hormonal therapies such as androgen deprivation therapy (ADT), Abiraterone, competitive androgen receptor blockers (Enzalutamide, for example), and chemotherapy are used in the metastatic setting [1]. New therapeutic modalities such as Androgen receptor signaling inhibitors (ARSIs), Poly ADP ribose polymerase (PARP) inhibitors and Lu-PSMA radioligand therapy are becoming more widely accessible [4]. Thus, in metastatic castration-sensitive prostate cancer, a current regime of treatment usually consists of a combination of ADT (such as GnRH agonists or antagonists) together with other novel drugs such as Abiraterone or Darolutamide [5]. Chemotherapy (such as docetaxel) may be utilized depending on the patient and disease characteristics.

Unfortunately, within about 11.7 months [6], patients often progress to a castration-resistant stage, reducing the effectiveness of systemic treatments [1].

Historically, local therapy to the prostate in the setting of metastatic disease was delivered solely for palliation [7]. In 2002, a SWOG retrospective analysis of a previous double randomized control trial on 1286 men with metastatic prostate cancer revealed a lower risk of mortality when local therapy (radical prostatectomy or radiation) was delivered [8]. Subsequently, efforts were made to further investigate the effect of local therapy for patients in the metastatic setting. Retrospective studies suggest that local radiation therapy improves overall survival in metastatic prostate cancer, especially in patients with better prognosis, less aggressive tumors, and better health [9,10,11,12,13,14]. However, prospective randomized control studies have not reproduced these positive results [7].

The STAMPEDE and HORRAD studies found no increase in overall survival with radiotherapy in addition to conventional treatment [7,15]. However, in a STAMPEDE sub-group with low metastatic burden (less than four metastases), radiotherapy improved 3-year survival to 81% vs. 73% in the control group (*p* = 0.007) [7]. In patients with high metastatic burden, there was no significant difference in 3-year survival: 54% in the control group vs. 53% in the RT group (*p* = 0.42). Moreover, the STOPCAP pooled meta-analysis of the two trials supported the finding of survival benefit in low-metastatic-burden disease [16].

The role of prostate radiation in high-metastatic-burden disease, according to the CHAARTED definition [17], is controversial, since the STAMPEDE trial showed no benefit for this group of patients. Yet, we hypothesis that there is a potential benefit for these patients with prostate radiation. Here we compare overall survival, progression-free survival, and symptom severity in patients receiving localized RT along with standard of care (SOC) systemic therapy versus SOC only.

## 2. Methods

### 2.1. Inclusion and Exclusion Criteria

Our study is a retrospective cohort study of high-metastatic-burden prostate cancer patients. We compare a group of patients who were treated with radiotherapy to the prostate (in addition to standard treatment) to those who received the standard treatment only. We also perform propensity score matching of patients from both groups accounting for different histopathological and clinical parameters.

Patients were included in this analysis if they had histologically confirmed adenocarcinoma of the prostate, had not previously received local therapy, and had four or more bone metastases on bone scintigraphy, PET-PSMA or CT imaging.

Tumors of any grade, T stage or N stage, were included. Patients who had received previous treatments for prostate cancer and were not newly diagnosed were excluded.

Patients were stratified according to treatment type:

- **RT Group**: Patients who received external beam radiation therapy (EBRT) targeting the prostate or prostate and pelvis within 6 months of diagnosis, with or without additional androgen deprivation therapy (ADT) or chemotherapy.
- **NRT Group**: Patients who received ADT, chemotherapy, or combinations of these treatments within 6 months of diagnosis.

The analysis included patients diagnosed and treated from 1 January 2004 to 31 December 2021. Out of 3234 prostate cancer patients, 524 had metastases at diagnosis (M1). Among them, 350 were treated with radiotherapy, and of these, 126 received EBRT targeting the prostate or prostate and pelvis within 6 months of diagnosis as first-line treatment. Patients with a low metastasis burden were excluded, resulting in the exclusion of 76 patients from the RT group and 124 from the NRT group.

Thus, 100 patients were included in our study, 50 in the RT group and 50 in the NRT group.

### 2.2. Statistical Methods, Clinical Variables and Definition of Outcomes

Patient information was collected from hospital medical records and anonymized before performing the analysis. Variables we included were basic demographics (age at diagnosis, ethnicity, and Charlson Comorbidity Index [CCI]), hospitalization times and durations, cancer staging and grading, treatment course, RT course, imaging after RT, vital status, clinical symptoms, PSA levels and cause of death. Prostate RT consisted of EBRT to the prostate with or without the pelvis.

Baseline characteristics between the RT and NRT groups and categorical variable were compared using statistical tests such as the chi-square χ^2^ test and Fischer’s exact test. Continuous Variables were compared using the Mann–Whitney test or *t*-test. Overall survival, progression-free time and time until symptom deterioration were estimated using the Kaplan–Meier method and univariate comparisons were performed using the log-rank test. The proportional hazards assumption was assessed for all covariates (age, year of diagnosis, Gleason score, ISUP [18], clinical tumor (T) stage, clinical nodal (N) stage, PSA level, different symptoms at diagnosis) and resulted in no statistical significance.

Propensity score matching (PSM) analysis was conducted to allow a more precise estimation of the treatment effect while mitigating selection bias. The PSM was computed utilizing a logistic regression model, and covariates included in the PSM model were as follows: CCI, Gleason score, T grade, N grade, age at diagnosis. A 1:1 matching method was used, resulting in 50 matched patients out of the original 100.

The primary objective for analysis was the comparison of Overall Survival (OS) from time of diagnosis between the RT and NRT groups. Secondary endpoints were progression-free survival (defined as time without Loco-Regional or Distant Progression), biochemical progression-free survival (defined as time between diagnosis and a PSA increase after the initiation of ADT of more than 50% of the lowest PSA value after the start of treatment, with a minimum of 1 ng/mL), Loco-Regional progression-free survival (defined as the time between diagnosis and local tumor recurrence or enlargement in tumor bed or seminal vesicles, occurrence of new nodes in the pelvis, retroperitoneum or under the iliac bifurcation), Distant progression-free survival (defined as time between diagnosis and distant metastasis occurrence on CT, MRI or bone scan or occurrence of new nodes over the iliac bifurcation). In addition, we analyzed time without symptom progression (defined as time between diagnosis and occurrence of new urinary incontinence or urinary retention or urethral catheter insertion or worsening of an existing symptom).

All the analyses were performed using SPSS 22.0, with all of them being two-sided and with a significance level of 0.05.

### 2.3. Ethical Considerations

The trial was conducted in accordance with the Declaration of Helsinki and approved by the local hospital Institutional Review Board.

## 3. Results

The entire cohort included 100 patients with metastatic prostate cancer who met the inclusion criteria, 50 in the RT group and 50 in the NRT group. Table 1 summarizes the clinicopathologic parameters of patients stratified by group. The median age was 74 years and the median follow-up time for the entire cohort was 26.48 months. In the RT group, forty-five patients received radiation to the prostate only and five to the prostate and pelvis due to nodal involvement (N1). There were no significant differences between the groups in the characteristics: Gleason score (*p* = 0.27), T stage (*p* = 0.53) or N stage (*p* = 0.53).

**Figure 1:**
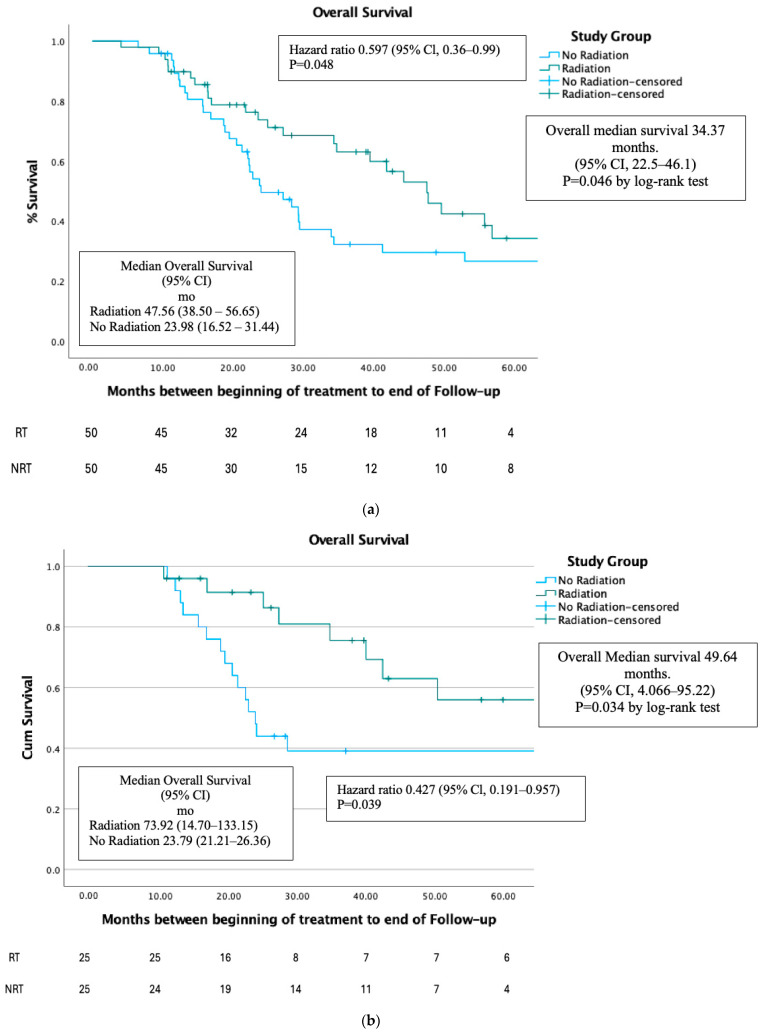
(**a**). OS before PSM. (**b**). OS after PSM.

Overall Survival was higher in the RT group compared to the NRT group (*p* < 0.046) (Figure 1a). For the entire cohort of patients, the median survival time was 34.37 months (95% confidence interval [CI] 22.5–46.1), for the RT group it was 47.56 months (95% CI 38.5–56.65), and for the NRT it was 23.98 months (95% CI 16.52–31.44).

**Figure 2:**
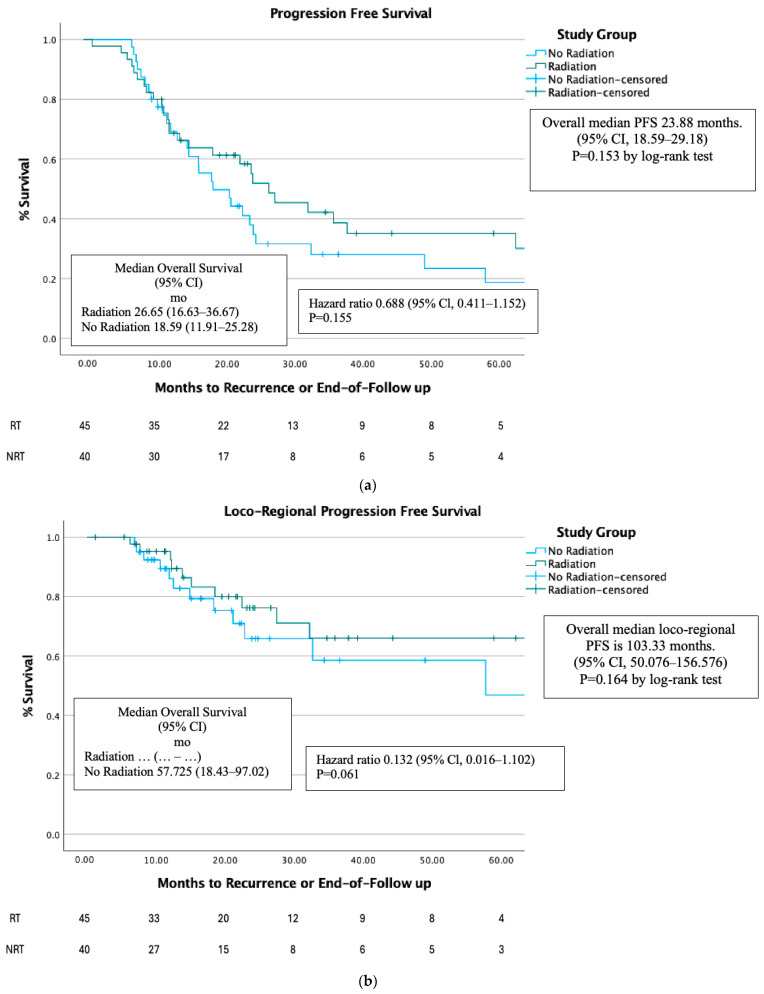

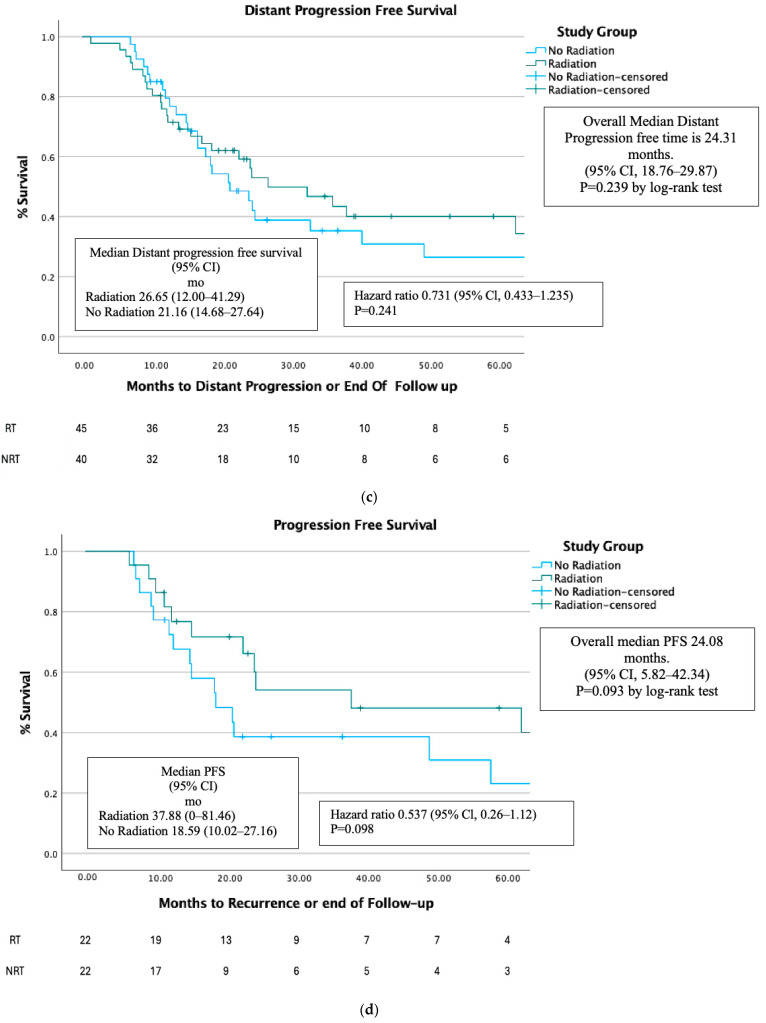

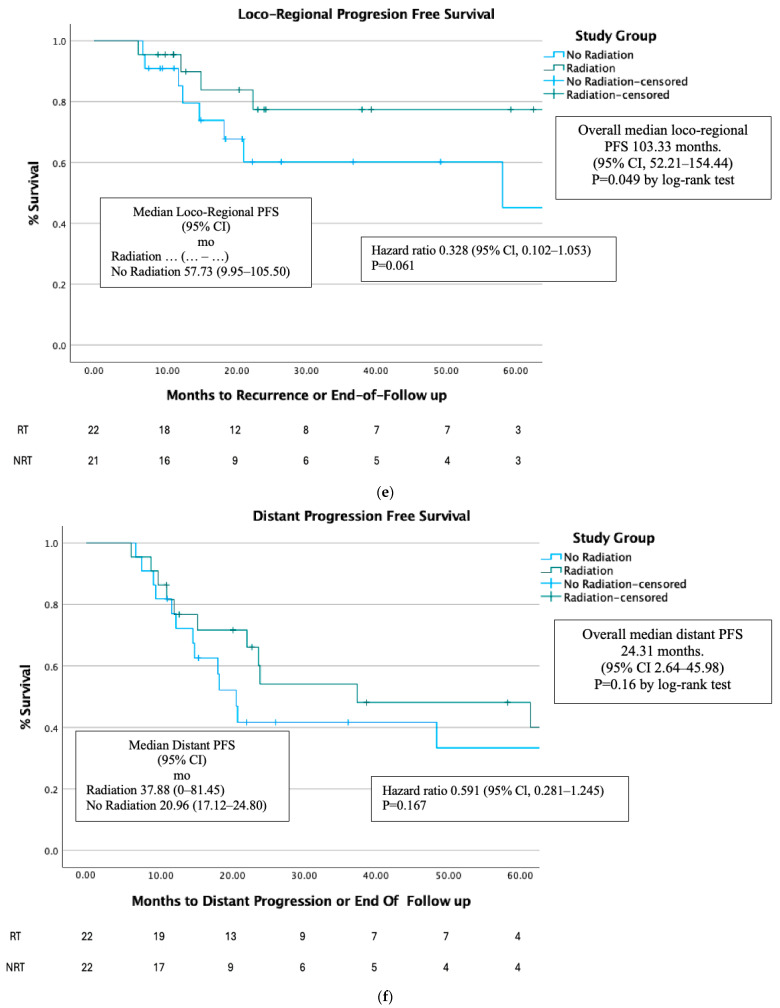
(**a**). PFS before PSM. (**b**). Loco-Regional PFS before PSM. (**c**). Distant PFS before PSM. (**d**). PFS after PSM. (**e**). Loco-Regional PFS after PSM. (**f**). Distant PFS after PSM.

There was no statistically significant difference in Progression-free survival (PFS) (Figure 2a), Loco-Regional PFS (Figure 2b) and Distant PFS (Figure 2c) between the RT group and NRT group.

**Figure 3:**
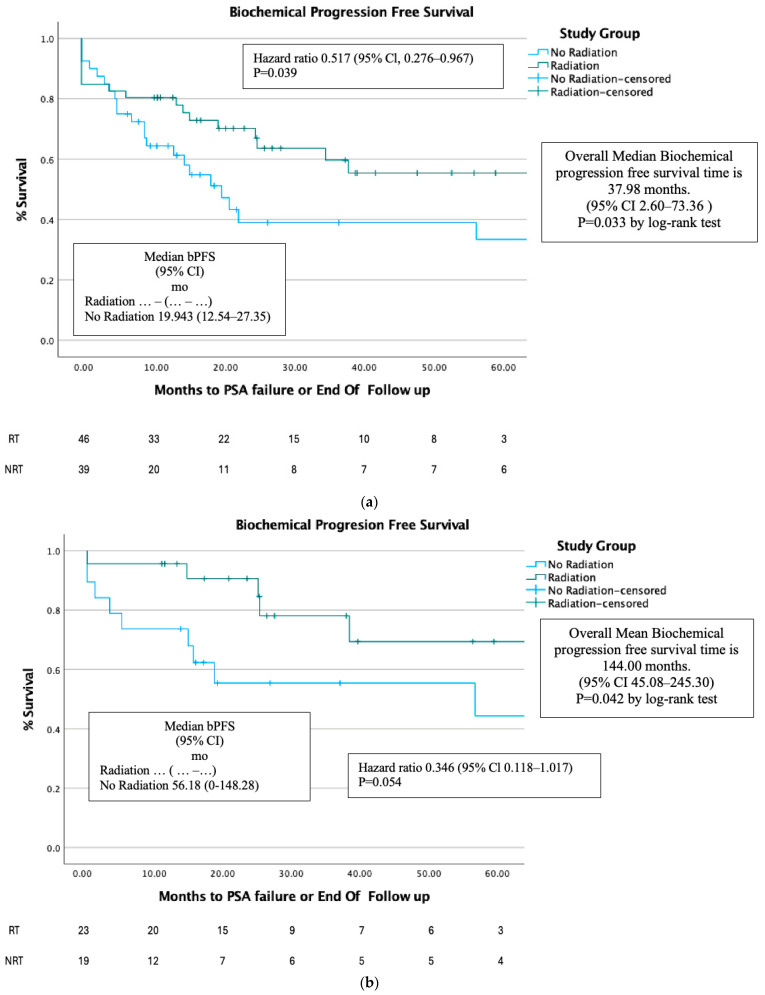
(**a**). bPFS before PSM. (**b**). bPFS after PSM.

Biochemical progression-free survival (bPFS) was higher in the RT group compared to the NRT group (*p* < 0.04) (Figure 3a). For the entire cohort of patients, the median survival time was 37.98 months (95% CI 2.60–73.36), while for the NRT group, it was 19.94 months (95% CI 12.54–27.35).

**Figure 4:**
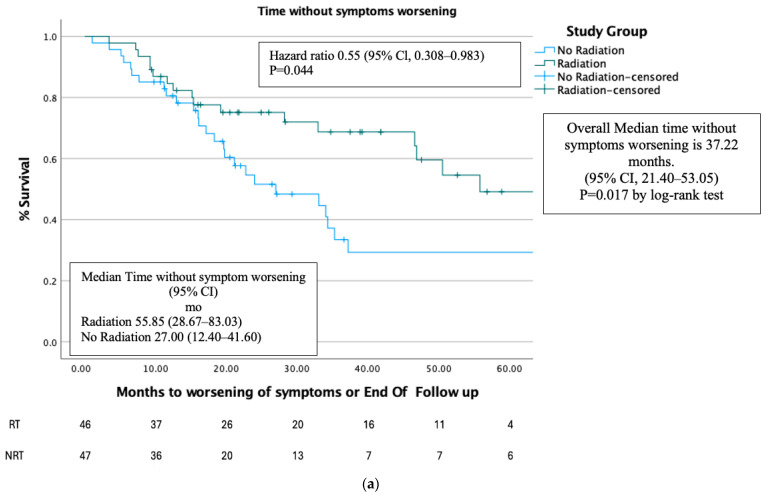

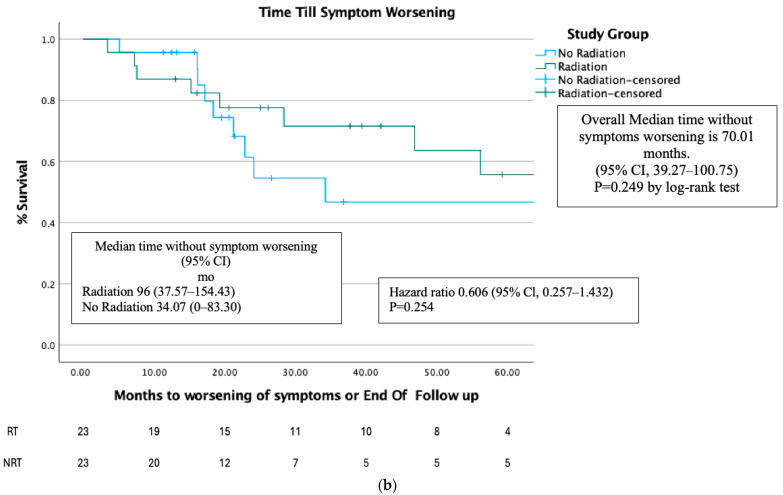
(**a**). Time without symptoms worsening before PSM. (**b**). Time without symptoms worsening after PSM.

Time without symptom worsening was higher in the RT group compared to the NRT group (*p* < 0.02) (Figure 4a). For the entire cohort of patients, the median time without symptoms worsening was 37.22 months (95% CI 21.40–53.05), for the RT group it was 55.85 months (95% CI 28.67–93.03), and for the NRT group it was 27.00 months (95% CI 12.40–41.60).

### Propensity Score Matching

After PSM, there were 25 patients left in each group. Table 2 summarizes patients’ clinicopathologically stratified characteristics. Median age was 71.5 and the median follow-up time for the cohort was 27.6. There were no significant differences between the groups in the following characteristics: Gleason score (*p* = 0.49 T stage (*p* = 0.49) or N stage (*p* = 0.36)).

Overall Survival was higher in the RT group compared to the NRT group (*p* < 0.04) after PSM. (Figure 1b) For the entire cohort of patients, the median survival time was 49.64 months (95% CI 4.066–95.22), for the RT group it was 73.92 months (95% CI 14.70–133.15), and for the NRT group it was 23.79 months (95% CI 21.21–26.36).

There was no statistically significant difference in PFS (Figure 2d) and distant PFS (Figure 2f) between the RT group and NRT group, but after PSM, Loco-Regional PFS was higher in the RT group compared to the NRT group (*p* < 0.05) (Figure 2e).

bPFS was higher in the RT group when compared to the NRT group (*p* < 0.04) (Figure 3b). For the entire cohort of patients, the median survival time was 111.55 months (95% CI 45.08–242.92), while for the NRT group it was 56.18 months (95% CI 0–148.28).

After PMS, there was no difference in time without symptom worsening in the RT group when compared to the NRT group (*p* < 0.25) (Figure 4b).

## 4. Discussion

Our study suggests that RT treatment on prostate cancer patients with significant metastatic burden is beneficial and extends overall survival. This finding is validated after performing PSM for different histopathological and clinical parameters, which allows us to control for those variables, mitigating potential bias and allowing a more precise estimation of the treatment effect [19]. Extensive experimental models had supported Paget’s “seed and soil” theory, which postulates that interactions between circulating tumor cells (the “seed”) and organ microenvironment (the “soil”) lead to metastasis formation in those distant organs [20]. The tumor supplies those circulating tumor cells but also might “prepare” the organs to accept those cells by creating a suitable microenvironment through endocrine effects. This implies that treating the primary tumor can delay the formation of distant metastasis [21].

Furthermore, emerging evidence shows that early palliative care in parallel to the anti-neoplastic treatments in different types of metastatic cancer, including prostatic cancer, can be beneficial for different clinical outcomes [22].

In prostate cancer, the ability of radiotherapy to increase overall survival of metastatic prostate cancer patients when added to systemic treatment was demonstrated in many retrospective studies [9,10,11,12,13,14]. The survival benefit associated with prostate RT was reported to be greater in patients with a better prognosis [8,9,10].

Despite positive findings in retrospective studies, prospective randomized control studies failed to demonstrate these results [7]. The STAMPEDE study, a large randomized control trial, found no increase in overall survival with radiotherapy added to conventional treatment compared to conventional treatment alone. In the STAMPEDE trial, the NRT group had a median survival time of 46 months, while the RT group survival was 48 months and not statistically different [7]. However, for patients with low metastatic burden (less than four metastases), radiotherapy improved prognosis [7]. Similarly, the HORRAD study found no survival difference between hormonal treatment with or without radiotherapy, though disease progression was faster in the former group, without statistical significance [15].

For the first time, we demonstrate increased overall survival in the high-burden group by adding RT to the accepted treatment (ADT with or without chemotherapy). In the post-PSM cohort, the RT group had a median survival of 73.92 months, compared to 23.79 months in the NRT group (*p* < 0.03).

Compared to the STAMPEDE trial, our NRT group had lower median survival (46 months vs. 23.98 months), while our RT group had similar median survival (48 months vs. 47.56 months). This could be due to differences in patient characteristics, such as a higher percentage of patients with T4 tumors in the STAMPEDE trial or access to newer systemic agents post-progression. Noteworthy is that both groups in our study were well balanced in terms of the clinical and histopathological parameters and causes of death (mostly prostate cancer-related). Furthermore, less than half of our NRT group received chemotherapy, mostly administered in proximity to the last follow-up. Our study’s favorable median survival suggests that using a strict cutoff of four metastases may not be optimal for determining RT benefits to the primary site.

Most other retrospective studies did not analyze the high-metastatic-burden subgroup. Cho et al. [14] in 2016 did analyze metastasis number (1 vs. 2–4 vs. 5) and sites (bone vs. other). They found improved OS in patients with only bone metastases, lower spread at diagnosis, and good performance status (3 year OS: 57% vs. 41% vs. 28%, respectively, *p* = 0.007) [14]. Loppenberg in 2017 did not analyze this subgroup but found better outcomes in patients with lower tumor risk or better general health [12].

The possibility that we found an improvement in overall survival (in contrast to the STAMPEDE and HORRAD trials) might be attributed to our cohort characteristics, which made them more responsive to radiotherapy (RT) when combined with systemic treatments. These characteristics could include specific genetic alterations, malignancy microenvironment factors or molecular markers that were not assessed or accounted for [23]. Given the information genomic classifiers contribute to patient management, this is a path for further investigation for prostate cancer radiation under this indication [24].

Interestingly, our results show that adding radiation to the prostate improves the delay in symptom worsening. In the full cohort, the median time without symptom worsening was longer in the RT group (55.85 months) compared to the NRT group (37.22 months). However, this difference was not seen after PSM. Past studies have not thoroughly analyzed quality of life (QOL) improvement after tumor RT in metastatic patients. Although the HORRAD trial collected QOL data, the analysis has not been published [15]. The delayed onset of symptom worsening we observed suggests a need for further studies comparing QOL between the groups.

Progression-free survival (PFS) was not significantly longer in the radiotherapy (RT) group in the full cohort or after PSM. The RT group had a median PFS time of 26.65 months, while the NRT group had 23.98 months (*p* < 0.15). Loco-Regional PFS was higher in the RT group (*p* < 0.05). After PSM, the RT group showed a larger numerical benefit with a median PFS of 37.88 months compared to 18.60 months for the NRT group. Despite this trend, the difference did not reach statistical significance (*p* = 0.09). A larger cohort could confirm the trend we observed in our PSM analysis.

The absence of statistically significant PFS improvement might be due to partial efficacy of the treatment against the cancer, as there are cancer cells that have already disseminated in the body. Additionally, cancer cells’ heterogeneity enables different properties in the tumor, and radiation-resistant clones of cells may lead to gradual disease progression [25]. A larger patient cohort might have yielded different results. This aligns with the STAMPEDE trial, where PFS was similar between RT and NRT groups (47% survival at 3 years) [7].

Biochemical progression-free survival was higher in the radiotherapy group in the full cohort (*p* = 0.03) and after PSM (*p* < 0.04). This was also shown in Cho et al. [14], where he showed that bPFS in 3 years was 69% in the RT group vs. 43% in the NRT group (*p* = 0.004). This can be attributed to the mechanism of the radiotherapy; it shrinks the local prostatic mass, lowering the PSA that is secreted from it [1].

Acknowledging our analysis’s limitations is crucial. Due to the retrospective design, there is a susceptibility to selection biases and unquantified variable imbalances. We were unable to control for certain factors such as performance status, smoking history, and co-morbidities beyond surrogate measures based on patient and disease status, including age, PSA level, Gleason score, T stage, N stage, and M stage. Additionally, full data on systemic therapy agents, duration of therapy, and salvage therapies were not available. This lack of comprehensive data might introduce confounding variables and limit our ability to adjust for their impact on outcomes. In addition, our sample size is relatively low and consists of patients concentrated in a single country and geographic area.

Therefore, while our analysis provides valuable insights, these limitations should be taken into consideration when interpreting the results. Further studies with prospective designs are necessary to address these limitations and provide a more robust understanding of the impact of radiotherapy in high-burden metastatic prostate cancer treatment.

## 5. Conclusions

Our study demonstrates longer overall survival in high-metastatic-burden prostate cancer patients when adding external beam radiation therapy to the prostate in addition to the standard treatment of choice. This finding persists after performing propensity score matching for different clinicopathologic parameters between the RT and NRT groups. The RT group in the entire cohort also demonstrated a reduction in progressing urinary symptoms and longer biochemical progression-free survival. These novel findings highlight the potential of radiotherapy as a viable treatment in this sub-group of prostate cancer patients. Despite the promising findings, the retrospective design introduces certain limitations; thus, there is a need for robust prospective studies with larger sample sizes to validate the findings.

## Figures and Tables

**Table 1:** Clinicopathologic parameters of full cohort.

Parameter	All Patients, N (%)	Radiotherapy—RT (%)	No Radiotherapy—NRT (%)	*p*-Value
Overall	100	50	50	
Gleason score				0.269
Gleason 6	1 (1.2)	1 (2)	0 (0)	
Gleason 7	9 (11)	3 (6.1)	6 (18.2)	
Gleason 8	23 (28)	14 (28.6)	9 (27.3)	
Gleason 9	30 (36.6)	21 (42.9)	9 (27.3)	
Gleason 10	19 (23.2)	10 (20.4)	9 (27.3)	
ISUP group				0.204
ISUP 1	1 (1.2)	1 (2)	0 (0)	
ISUP 2	5 (15.2)	1 (2)	6 (7.3)	
ISUP 3	1 (3.0)	2 (4.1)	3 (3.7)	
ISUP 4	9 (27.3)	14 (28.6)	23 (28)	
ISUP 5	18 (54.5)	31 (63.3)	49 (59.8)	
T stage				0.529
T1	5 (6.1)	2 (4.4)	3 (8.1)	
T2	10 (12.2)	6	4	
T3	56 (68.3)	29	27	
T4	11 (13.4)	8	3	
N stage				0.22
N0	22 (28.6)	9 (22.5)	13 (35.1)	
N1	55 (71.4)	31 (77.5)	24 (64.9)	
M stage				1
M1	100 (100)	50 (100)	50 (100)	
Type of non-radiotherapy treatment				0.689
Only ADT used	51 (51)	24 (48)	27 (54)	
ADT + Chemothreapy used	49 (49)	26 (52)	23 (46)	
Ethnicity				0.548
White	49 (49)	26 (52)	23 (28)	
Middle Eastern	51 (51)	24 (48)	27 (54)	
Parameter	All patients, Median	Radiotherapy Median	No Radiotherapy Median	*p*-value
Median follow-up time	26.48	34.36	23.04	0.494
Median Age	74	73.87	74.5	0.825
Charlson Comorbidity index Score	9.91	9.88	9.94	0.856

**Table 2:** Clinicopathologic parameters of cohort after PMS.

Parameter	All Patients, N (%)	Radiotherapy—RT (%)	No Radiotherapy—NRT (%)	*p*-Value
Overall	50	25	25	
Gleason score				0.488
Gleason 6	0 (0)	0 (0)	0 (0)	
Gleason 7	3 (6)	2 (4)	1 (2)	
Gleason 8	18 (36)	9 (18)	9 (18)	
Gleason 9	17 (34)	9 (18)	8 (16)	
Gleason 10	10 (20)	4 (8)	6 (12)	
ISUP group				0.494
ISUP 1	0 (0)	0 (0)	0 (0)	
ISUP 2	2 (4)	1 (2)	1 (2)	
ISUP 3	1 (2)	1 (2)	0 (0)	
ISUP 4	18 (36)	9 (18)	9 (18)	
ISUP 5	27 (54)	13 (26)	14 (28)	
T stage				0.491
T1	1 (2)	1 (2)	0 (0)	
T2	4 (8)	3 (6)	1 (2)	
T3	36 (72)	17 (34)	19 (38)	
T4	3 (6)	1 (2)	2 (4)	
N stage				0.359
N0	11 (22)	6 (12)	5 (10)	
N1	30 (60)	15 (30)	15 (30)	
M stage				0.5
M1	50 (100)	25 (100)	25 (100)	
Type of non-radiotherapy treatment				0.243
Only ADT used	24 (48)	13 (26)	11 (22)	
ADT + Chemothreapy used	25 (50)	11 (22)	14 (28)	
Ethnicity				0.391
White	25 (50)	13 (26)	12 (24)	
Middle Eastern	25 (50)	12 (24)	13 (26)	
Parameter	All patients, Mean	Radiotherapy Mean	No Radiotherapy Mean	*p*-value
Median follow-up time	27.6	39.2	23.78	0.488
Mean Age	70.74	68.76	72.72	0.068
Charlso Comorbidity index Score	9.64	9.4	9.88	0.128

## Data Availability

The paper shares all of the data used for analyzing the results.
